# Understanding university teachers’ continuance of an AI teaching assistant: an integrated TTF–TAM–ECM model in higher education

**DOI:** 10.3389/fpsyg.2026.1765263

**Published:** 2026-03-16

**Authors:** Zhihan Liu, Sha Cao, Jingwei Zhang, Thanawan Phongsatha, Satha Phongsatha

**Affiliations:** 1 School of Foreign Studies, University of Science and Technology Liaoning, Anshan, China; 2 Graduate School of Business and Advanced Technology Management, Assumption University, Bangkok, Thailand; 3 Graduate School of Human Sciences, Assumption University, Bangkok, Thailand

**Keywords:** continuance intention, Expectation-Confirmation Model, task-technology fit, teacher adoption, Technology Acceptance Model

## Abstract

**Introduction:**

The rapid integration of artificial intelligence (AI) into higher education is reshaping teachers’ work, yet limited evidence addresses teachers’ post-adoption experiences with AI teaching assistants. This study examines university English teachers’ continuance use of the Superstar AI Assistant by integrating the Technology Acceptance Model, Expectation–Confirmation Model, and Task–Technology Fit.

**Methods:**

Survey data from 248 teachers who used the AI assistant for one semester were collected and analyzed using Structural Equation Modeling with bootstrapped mediation analyses.

**Results:**

Task–technology fit strongly predicts perceived ease of use, confirmation, satisfaction, and behavioral intention, whereas its direct effects on perceived usefulness and actual use are non-significant. Perceived usefulness, ease of use, and confirmation significantly enhance satisfaction, and behavioral intention is the primary driver of actual use. Multiple significant indirect pathways were identified through mediation analyses.

**Discussion:**

The study advances post-adoption theory in AI-supported teaching and highlights implications for teacher professional development, AI system design, and institutional digital transformation.

## 1 Introduction

Artificial intelligence (AI) is rapidly reshaping higher education, transforming how teachers plan, deliver, and evaluate instruction. International bodies such as UNESCO and OECD highlight AI’s potential to enhance instructional efficiency, personalize learning, and reduce administrative workload (Arruda and Arruda, 2024). In universities, AI tools increasingly support lesson preparation, content curation, classroom interaction, assessment, student monitoring, and academic decision-making, making teachers’ sustained engagement with AI a critical research priority.

In China, national “Smart Education” initiatives have driven large-scale integration of AI-based platforms across universities (Liu et al., 2024). The Superstar Teaching Platform—especially its embedded Superstar AI Assistant—has become widely adopted for English teaching and is distinctive in its comprehensive, multi-function design. Unlike single-task generative AI tools such as ChatGPT, the Superstar AI Assistant supports the entire instructional cycle, embedding AI deeply into teachers’ daily professional routines. This provides an ideal context for examining authentic, post-adoption teacher experiences beyond initial novelty effects.

Despite AI’s rapid diffusion, empirical research on teachers’ post-adoption use remains limited. Existing scholarship disproportionately emphasizes students’ acceptance, AI ethics, design frameworks, and institutional readiness (Ouyang et al., 2022; Holmes et al., 2022), while comparatively little is known about how teachers evaluate and continue using AI in real instructional settings (Ifenthaler and Yau, 2020; Choi et al., 2026). Moreover, many studies rely on single-theory models such as TAM or UTAUT, overlooking post-adoption constructs—confirmation, satisfaction, and task–technology alignment—central to understanding sustained technology engagement (Zawacki-Richter et al., 2019). Consequently, key questions remain underexplored: Which psychological and experiential mechanisms drive teachers’ intention to continue using AI? And how do AI tools shape teaching behaviors after extended use?

The evidence base is especially thin in non-Western higher education contexts. Cultural, institutional, and pedagogical norms in East Asia influence technology use in unique ways (Li, 2025), yet rigorous analyses of teachers’ AI continuance—particularly regarding multifunctional AI assistants used across the full teaching cycle—are scarce. This gap limits global understanding of teacher–AI interaction in authentic environments where AI is positioned as an ongoing pedagogical partner.

To address these gaps, this study investigates university English teachers’ post-adoption perceptions and continuance use of the Superstar AI Assistant using an integrated framework that combines the Technology Acceptance Model (TAM), the Expectation–Confirmation Model (ECM), and Task–Technology Fit (TTF). This integration enables examination of cognitive beliefs (perceived usefulness, perceived ease of use), experiential judgments (confirmation, satisfaction), and task alignment (task–technology fit), and how these jointly shape continuance intention and actual use. Structural Equation Modeling (SEM), complemented by supplementary qualitative reflections, is used to test both direct and mediating relationships within this post-adoption context.

This study offers three contributions. Theoretically, it advances teacher-centered AI research by validating a unified TAM–ECM–TTF model for AI-supported university teaching. Empirically, it provides rare post-adoption evidence from Chinese higher education, showing how multifunctional AI assistants influence teachers’ perceptions and sustained use. Practically, the results offer guidance for designing and supporting AI systems that meaningfully align with teachers’ professional work and institutional teaching goals.

## 2 Literature review

Artificial intelligence (AI) is now woven into university teaching, reshaping teachers’ planning, instruction, assessment, and decision-making. As AI becomes embedded across the instructional cycle, teachers’ cognitive perceptions, experiential evaluations, and task alignment have become central to understanding sustainable AI integration.

### 2.1 Artificial intelligence in teaching and teacher workflows

AI systems increasingly automate routine instructional tasks and provide feedback, resource recommendations, analytics, and conversational assistance (Holmes et al., 2022; Ng et al., 2023; Lee and Moore, 2024; Uygun, 2024). While such tools can shift teachers’ work toward more design-oriented practice, they also raise concerns related to autonomy, trust, cognitive load, and identity (Zawacki-Richter et al., 2019; Zuo et al., 2025). Teachers ultimately judge AI by its contribution to pedagogical goals and by whether it supports—not replaces—their expertise (Malakul, 2025). Although AI has demonstrated value in assessment, personalization, and analytics (Ifenthaler and Yau, 2020; Liang et al., 2025), research on post-adoption experiences—confirmation, satisfaction, continuance intention, and sustained use—remains limited (Xue et al., 2025). Responding to calls for teacher-centered, context-embedded work (Zuo et al., 2025; Liang et al., 2025), the present study treats AI as part of teachers’ professional ecosystem and focuses on their long-term interaction with an institutional AI assistant.

### 2.2 Technology Acceptance Model (TAM) in AI-supported teaching

The Technology Acceptance Model (TAM) continues to explain teachers’ adoption of educational technologies, with Perceived Usefulness (PU) and Perceived Ease of Use (PEOU) predictive of intention (Davis, 1989; Wang et al., 2025; Chavarnakul et al., 2024). In AI-supported teaching, PU captures perceived gains in instructional quality, workload reduction, and pedagogical reasoning (Holmes et al., 2022; Song, 2025), while PEOU reflects intuitive interaction, transparency, and cognitive effort (Zawacki-Richter et al., 2019; Malakul, 2025; Uygun, 2024).

Teacher-focused studies adapt TAM to professional concerns, noting that pedagogical usefulness and cognitive effort are particularly salient (Lin et al., 2025). Even where teachers express reservations about trust or autonomy, PU and PEOU remain strong predictors of both adoption and continuance (Zuo et al., 2025; Xue et al., 2025). Recent work shows TAM performs best when integrated with post-adoption variables—confirmation, satisfaction, and task alignment (Chavarnakul et al., 2024; Liang et al., 2025). In this study, PU and PEOU reflect teachers’ evaluations of the Superstar AI Assistant after extensive real-world use and are expected to shape confirmation, satisfaction, and continuance intention.

### 2.3 Expectation–Confirmation Model (ECM) and technology continuance in teaching

The Expectation–Confirmation Model (ECM) explains post-adoption behavior by positioning confirmation, satisfaction, and perceived usefulness as central determinants of continuance (Bhattacherjee, 2001). As teachers interact with AI across multiple teaching cycles, confirmation—whether AI performance meets expectations—becomes a key driver of satisfaction and continued use (Hong et al., 2006; Ng et al., 2023; Li et al., 2022). In AI contexts, confirmation concerns reliability, accuracy, workload reduction, and pedagogical value (Holmes et al., 2022; Zuo et al., 2025).

Satisfaction is especially predictive in routinised AI use, shaped by trustworthiness, transparency, and alignment with professional values (Malakul, 2025; Liang et al., 2025). PU also expands to reflect AI’s support for reasoning, feedback, and personalized instruction (Lin et al., 2025). Empirical work consistently shows that ECM explains post-adoption behavior more effectively than adoption-only models, particularly when combined with TAM and TTF (Du and Phongsatha, 2025; Chavarnakul et al., 2024). The present study uses ECM to examine how teachers’ confirmation and satisfaction with the Superstar AI Assistant shape continuance intention and use after one semester of daily interaction.

### 2.4 Task–technology fit (TTF) and alignment between AI tools and teaching workflows

Task–Technology Fit (TTF) holds that technology produces higher use and performance benefits when its functionalities match task requirements (Goodhue and Thompson, 1995). This is particularly relevant in AI-enhanced teaching, where tools support complex instructional, assessment, and classroom management tasks (Malakul, 2025).

AI-based functions—automated grading, analytics, resource generation, adaptive recommendations—map directly onto core teaching responsibilities (Alwakid et al., 2025; Zuo et al., 2025). When TTF is high, teachers are more likely to integrate AI into daily routines. Evidence shows that TTF predicts PU, confirmation, and satisfaction (Li et al., 2022; Malakul, 2025), and that misalignment—burdensome or inaccurate AI output—reduces satisfaction despite high ease of use (Xue et al., 2025). Conversely, pedagogically meaningful AI support enhances TTF and promotes continuance (Lin et al., 2025; Liang et al., 2025).

Because the Superstar AI Assistant is used across the entire instructional cycle, TTF is critical for examining whether system functions genuinely support teachers’ task demands and how such alignment influences perceptions, satisfaction, and sustained use.

### 2.5 Integrating TAM, ECM, and TTF for post-adoption behavior in AI-supported teaching

Single-theory models cannot fully explain sustained AI use, as post-adoption behavior involves intertwined cognitive, experiential, and task-level processes. Scholars therefore increasingly integrate TAM, ECM, and TTF to capture technology continuance more holistically (Larsen et al., 2009; Lin et al., 2025; Malakul, 2025).

TAM contributes PU and PEOU (Venkatesh and Davis, 2000; Xue et al., 2025), ECM adds confirmation and satisfaction (Bhattacherjee, 2001), and TTF explains alignment with instructional tasks (Alwakid et al., 2025). Empirical work shows TTF shapes PU and confirmation; PU and confirmation shape satisfaction; and satisfaction predicts continuance (Li et al., 2022; Malakul, 2025). Studies also indicate hybrid models outperform single-model frameworks in predicting sustained AI engagement (Lin et al., 2025). This study adopts a unified TTF–TAM–ECM framework to examine teachers’ continuance behavior with the Superstar AI Assistant.

### 2.6 Summary, research gap, and conceptual framework

Prior research highlights AI’s potential to support preparation, instruction, assessment, and professional decision-making (Ifenthaler and Yau, 2020; Holmes et al., 2022; Lund and Wang, 2023). However, the field remains dominated by early-stage adoption studies, with limited attention to teachers’ post-adoption experiences after sustained use. Key gaps include:

1. Theoretical fragmentation: TAM, ECM, and TTF are rarely integrated, despite teachers’ continuance depending simultaneously on usefulness/ease (TAM), confirmation/satisfaction (ECM), and task alignment (TTF) (Or, 2025; Wang et al., 2025).
2. Limited evidence from comprehensive AI systems: Most studies examine isolated or experimental tools, not institutional AI assistants embedded across entire teaching workflows.
3. Insufficient longitudinal exposure: Many studies examine teachers after limited use, weakening the validity of post-adoption constructs (Hong et al., 2006).
4. Sparse mediation analyses: Few studies examine how cognitive, experiential, and task-fit mechanisms jointly shape behavioral intention and actual use.

This study addresses these gaps by investigating university English teachers’ post-adoption cognition and continuance behavior after a full semester of daily use of the Superstar AI Assistant. Using an integrated TTF–TAM–ECM model, the study examines how task alignment, cognitive evaluations, and experiential judgments shape continuance intention and use, complemented by qualitative reflections.

The study is guided by four research questions:

RQ1: How do teachers evaluate the usefulness, ease of use, confirmation, satisfaction, and task–technology fit of the AI assistant after extended use?

RQ2: What are the direct structural relationships among TTF, PEOU, PU, confirmation, satisfaction, behavioral intention, and actual use?

RQ3: Which mediating pathways explain how cognitive and experiential evaluations shape continuance intention and use?

RQ4: How do teachers’ qualitative reflections contextualize the mechanisms identified in the structural model?

**Figure 1:**
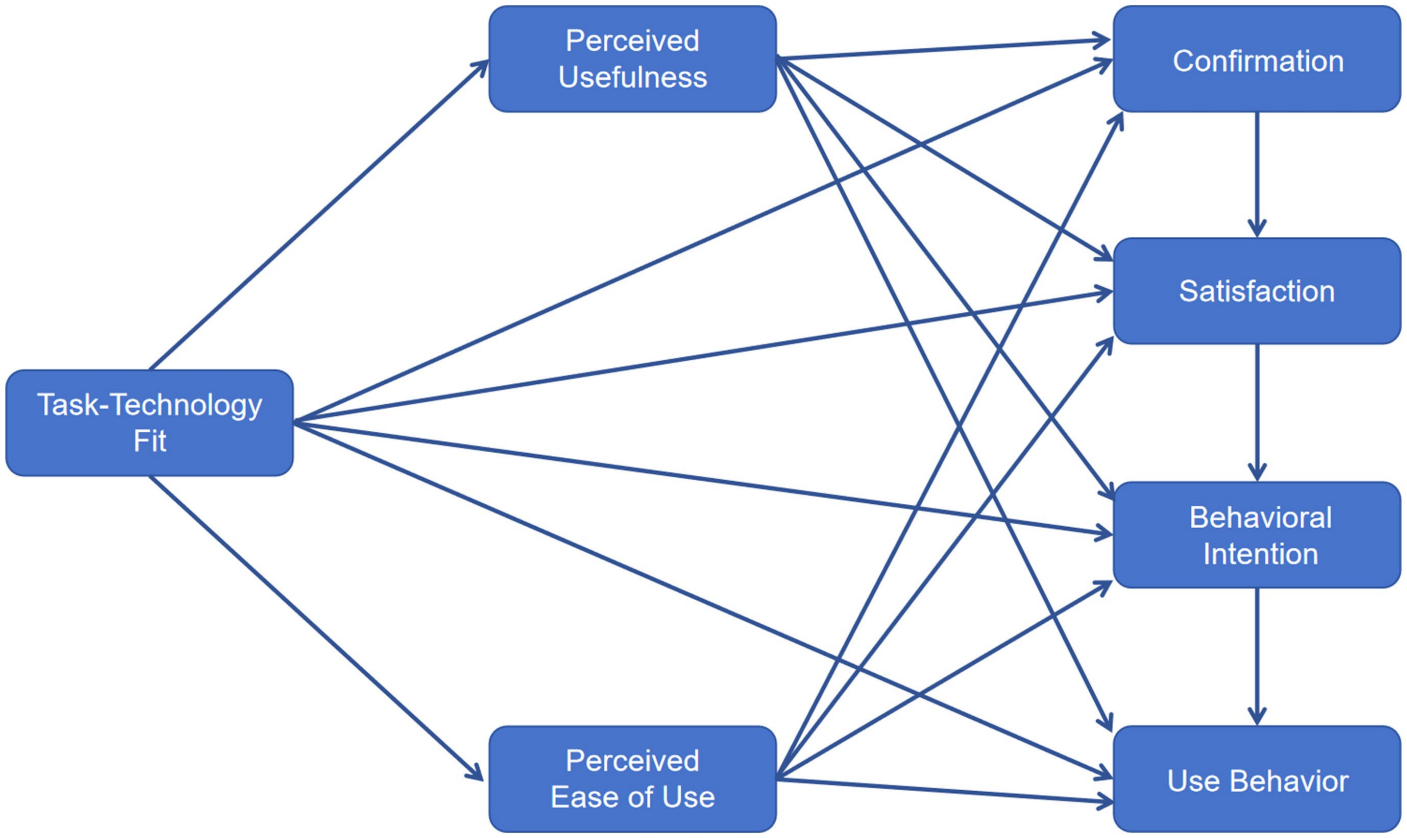
Conceptual framework.

Figure 1 presents the conceptual framework synthesizing TTF, TAM, and ECM into a unified structural model.

## 3 Methodology

This study adopted a quantitative-dominant design grounded in TAM, ECM, and TTF, supplemented by one optional open-ended item to enrich interpretation (Ning et al., 2024; Ifenthaler and Yau, 2020).

### 3.1 Research design

A cross-sectional explanatory design was used to test an integrated TTF–TAM–ECM model via Structural Equation Modeling (SEM), which is appropriate for examining multidimensional latent constructs and complex causal relations in educational technology research (Hair et al., 2019; Kline, 2023).

**Figure 2:**
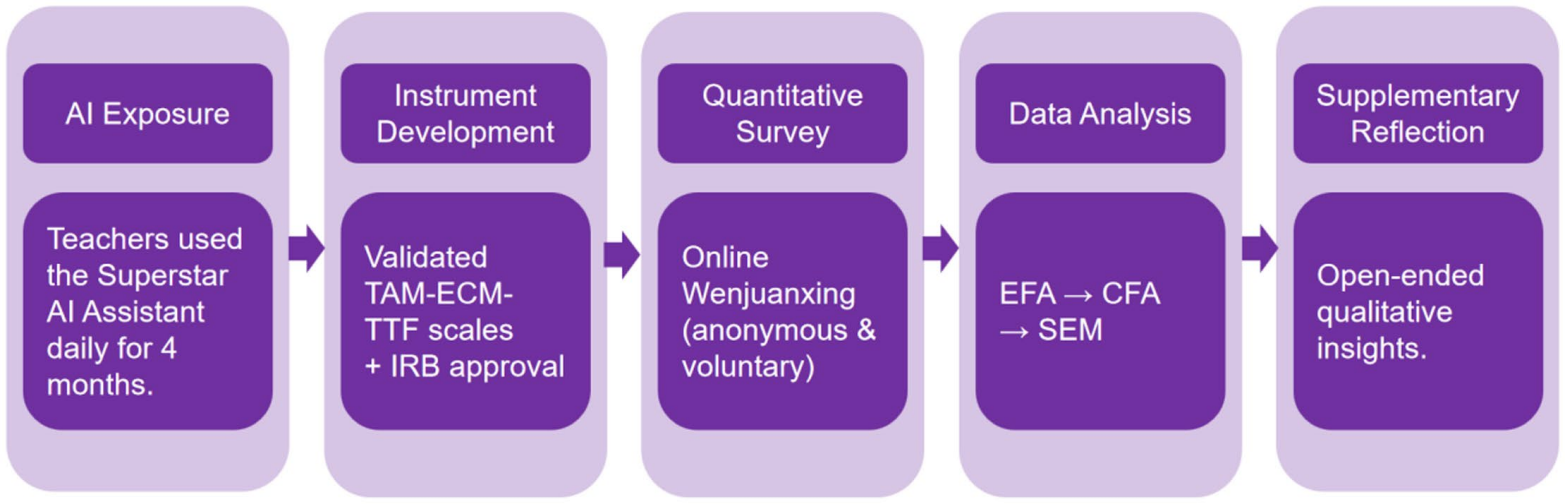
Study procedure and research design.

The study proceeded through five sequential phases: (1) 4 months of AI exposure, (2) instrument adaptation and pilot testing, (3) cross-sectional online survey, (4) EFA, CFA, and SEM (including mediation), and (5) descriptive analysis of qualitative reflections. Figure 2 summarizes this procedure.

A one-semester exposure period ensured that teachers had substantial experience with the assistant across preparation, in-class work, assessment, and student management, as recommended for post-adoption research (Bhattacherjee, 2001; Hong et al., 2006). All items used a 5-point Likert scale (1 = strongly disagree, 5 = strongly agree), consistent with SEM practice (Byrne, 2016).

The survey also included a single open-ended prompt inviting brief comments on using the AI assistant. These reflections were not formally coded but were used to clarify mechanisms underlying confirmation, satisfaction, and continuance, following “qualitative enhancement” logic in mixed-methods work (Creswell and Plano Clark, 2018).

Data were collected via Wenjuanxing, a secure online platform widely used in Chinese social science research. Participation was voluntary and anonymous; ethical procedures are described in Section 3.5.

### 3.2 Context of the study

The study was conducted at a provincial public university in Northeast China, where the Superstar Teaching Platform and its embedded Superstar AI Assistant have been systematically implemented in English language teaching as part of a digital transformation agenda aligned with national “Smart Education” policy (Liu et al., 2024).

The Superstar AI Assistant functions as a multi-purpose pedagogical support system, offering AI-driven lesson planning, resource recommendation, in-class interaction tools, automated grading, personalized feedback, early-warning analytics, and basic research support. The university provides workshops and technical training to strengthen teachers’ digital competence and ensure proficient use.

All respondents were full-time English teachers who had used the assistant for at least one semester (4 months), ensuring sufficient exposure for valid measurement of confirmation, satisfaction, and continuance intention (Bhattacherjee, 2001; Hong et al., 2006). Use was strongly encouraged and tightly integrated with the institution’s digital infrastructure, so participants were active, regular users, providing high ecological validity and capturing authentic post-adoption behavior rather than experimental trial use.

### 3.3 Participants and sampling

The target population comprised all university English teachers at the institution who had integrated the Superstar AI Assistant into their daily work. A census-based convenience approach was employed: all eligible teachers were invited via an official online notice. This strategy is suitable for bounded, well-defined institutional populations in technology-adoption research (Creswell and Plano Clark, 2018; Ifenthaler and Yau, 2020).

A total of 248 valid responses were retained after listwise deletion of incomplete questionnaires and removal of duplicates. The sample included teachers of varying age, rank, and course responsibilities, broadly matching the composition of the English faculty.

Sample adequacy for SEM was assessed using Soper (2020) SEM Sample Size Calculator. Assuming an expected effect size of 0.30, seven latent variables, 38 observed variables, *α* = 0.05, and power = 0.90, the recommended minimum was 210 cases.

**Figure 3:**
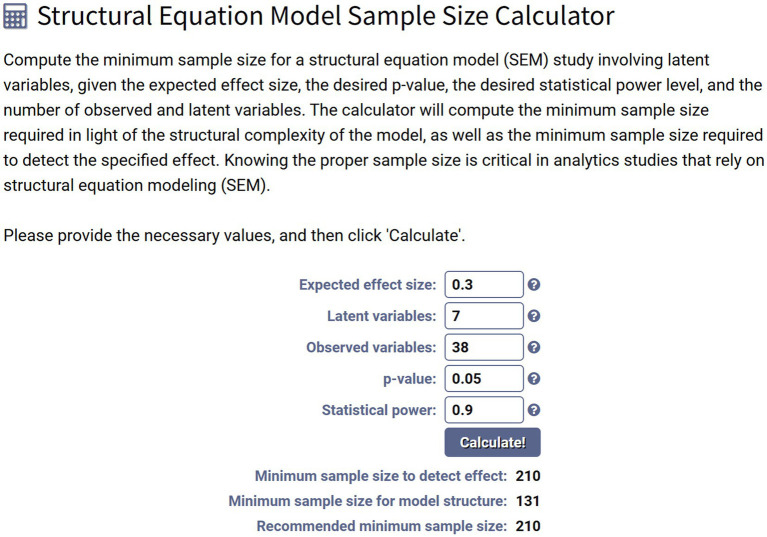
Sample size estimation for the structural equation model.

As shown in Figure 3, the achieved *N* = 248 exceeded this threshold, satisfying power and model-complexity requirements and aligning with guidance on sample-to-parameter ratios in SEM (Hair et al., 2019; Kline, 2023; Wolf et al., 2013).

### 3.4 Instruments and measures

A structured questionnaire measured seven latent constructs derived from TAM, ECM, and TTF, all on a 5-point Likert scale. Full operationalization, item wording, and sources are documented in Appendix A: Operationalization of Constructs.

#### 3.4.1 Construct operationalization

Seven multi-item variables were included:

- Task–Technology Fit (TTF) (6 items), adapted from Goodhue and Thompson (1995) and Dishaw and Strong (1999), capturing the degree to which the assistant’s functionalities match teachers’ instructional tasks.
- Perceived Usefulness (PU) (6 items), from Davis (1989) and Venkatesh and Davis (2000), assessing perceived performance enhancement attributable to the AI assistant.
- Perceived Ease of Use (PEOU) (4 items), from Davis (1989), reflecting perceived effort required to use the system.
- Confirmation (C) (4 items), from Bhattacherjee (2001), measuring consistency between expectations and actual experience.
- Satisfaction (S) (6 items), adapted from Bhattacherjee (2001), Oliver (2014), and Wixom and Todd (2005), capturing affective evaluations of the system.
- Behavioral Intention (BI) (6 items), based on UTAUT (Venkatesh et al., 2003) and ECM, assessing intention to continue use.
- Use Behavior (UB) (6 items), from UTAUT and ECM, indicating self-reported frequency and depth of use.

Scale adaptation followed best practices for contextualizing validated measures in educational technology settings (Venkatesh et al., 2012). Appendix A specifies each item’s theoretical origin and its tailoring to AI-supported teaching.

#### 3.4.2 Content validity

Content validity was assessed using the Content Validity Index (CVI) with four expert reviewers, following Polit and Beck (2006). Experts were required to hold a doctorate, possess extensive educational expertise, and be serving university professors. Each rated item relevance on a 4-point scale (Davis, 1992).

Item-level CVI (I-CVI) was calculated as the proportion of experts assigning ratings of 3 or 4. All items obtained I-CVI = 1.00. The scale-level CVI (S-CVI), calculated as the mean I-CVI, was therefore also 1.00, surpassing recommended benchmarks (Polit and Beck, 2006; Waltz et al., 2016). This indicates that the instrument adequately represents the content domain of AI-assisted teaching adoption.

#### 3.4.3 Reliability and pilot testing

A pilot study with 37 English teachers from the same institution was conducted to assess reliability. Cronbach’s alpha coefficients ranged from 0.910 to 0.983 across constructs (Table 1), well above the 0.70 criterion and within the “excellent” range for SEM applications (Gliem and Gliem, 2003; Byrne, 2016). These results supported proceeding to full-scale data collection.

**Table 1:** Pilot reliability results.

Construct	Cronbach’s alpha
Perceived usefulness	0.944
Confirmation	0.980
Task–technology fit	0.957
Perceived ease of use	0.926
Satisfaction	0.910
Behavioral intention	0.926
Use behavior	0.983

#### 3.4.4 Language and translation consideration

All participants were university English teachers with sufficient English proficiency; therefore, no translation or back-translation was required, avoiding cross-linguistic measurement error (Brislin, 1980).

### 3.5 Data collection and ethical consideration

Data were collected via a Wenjuanxing online questionnaire between 1 and 20 July 2025. A census-based convenience strategy invited all English teachers who had used the Superstar AI Assistant for at least one semester, consistent with institutional technology-adoption studies in higher education (Creswell and Plano Clark, 2018; Ifenthaler and Yau, 2020). The survey link was distributed by the academic administration office.

On accessing the survey, participants encountered an introductory page outlining the study purpose, voluntary participation, absence of incentives, and guarantees of anonymity and confidentiality. Proceeding to the questions signified informed consent, in line with guidelines for online research (Fink, 2017). The survey required approximately 10 min and consisted of demographics, the main scales, and one optional open-ended item.

In total, 248 valid responses were retained after listwise deletion of incomplete entries and removal of duplicates. No identifying information was collected. Data collection adhered to GDPR-aligned principles of data minimisation, confidentiality, and restricted access (European Union, 2016). Raw data were stored on the principal investigator’s encrypted laptop, accessible only to the researcher, consistent with best-practice recommendations (American Educational Research Association, 2011).

Ethical approval was obtained from the Ethics Review Committee of the School of Foreign Studies, University of Science and Technology Liaoning (IRB Approval Number: 202503-01, effective 1 March 2025). Procedures followed institutional and national guidelines and the Declaration of Helsinki. The study involved no foreseeable risk, as it focused solely on teachers’ perceptions of AI in professional contexts. Optional qualitative reflections were anonymous and used only to enrich interpretation, consistent with recommendations for supplementary qualitative components in quantitative-dominant designs (Creswell and Plano Clark, 2018).

Of the 248 valid respondents, all provided written responses to this item. These qualitative data were reviewed descriptively rather than coded for themes. The first author reviewed the data iteratively to identify recurring observations that “resonated” with the constructs from the quantitative data (e.g., task technology fit, confirmation, satisfaction, perceived usefulness). This procedure aligns with “qualitative enhancement” strategies for conducting mixed-methods research where quantitative results predominate (Creswell and Plano Clark, 2018; Sandelowski, 2000).

The quotes selected for analysis met three criteria: (a) clarity and expression, (b) relevance to key constructs in the structural model, and (c) ability to shed light on processes underlying constructs of confirmation, satisfaction, and continuance. No inter-rater reliability was calculated for the selected quotes, which was also consistent with the use of this data for illustrative purposes rather than analysis.

### 3.6 Data analysis

Data analysis followed a staged SEM procedure. All analyses were conducted in Jamovi, with Excel used for preliminary screening.

First, data were checked for missing values, outliers, and univariate normality. Likert items were treated as continuous, following SEM conventions (Kline, 2023). An Exploratory Factor Analysis (EFA) using minimum residual extraction with oblimin rotation examined the underlying factor structure of the adapted scales.

Next, Confirmatory Factor Analysis (CFA) tested the measurement model. Internal consistency was assessed using Cronbach’s alpha and Composite Reliability; convergent validity using Average Variance Extracted (AVE); and discriminant validity using the Fornell–Larcker criterion (Fornell and Larcker, 1981; Hair et al., 2019).

After establishing measurement adequacy, SEM was employed to examine the hypothesized relationships among TTF, PEOU, PU, C, S, BI, and UB. Model fit was evaluated using RMSEA, CFI, TLI, and SRMR. Indirect effects were tested via bootstrapped mediation analysis to obtain robust estimates of mediated pathways (Hayes and Rockwood, 2020).

Finally, the brief open-ended reflections were reviewed descriptively to illuminate patterns in the quantitative results. These comments were used to contextualize and exemplify the structural model rather than to constitute a separate qualitative study.

### 3.7 Procedural and statistical controls for common method bias

Since all the data was collected at a single time point via self-reporting, common method bias (CMB) was a possible threat to the results (Podsakoff et al., 2003). Several strategies were used to mitigate this issue during the design and administration of the survey. First, respondents were assured anonymity to reduce the effects of socially desirable answering. Second, the order of construct items was randomized to reduce consistency motifs. Third, scales with different response scales and formats were used to reduce method effects.

Several *post hoc* statistical analyses were also carried out to assess the presence of CMB. First, Harman’s single factor test was carried out to see if a majority of variance was explained by a single factor. Since the first factor explained only 38.7% of the variance (which was less than the 50% cutoff), this test suggested that there was little evidence for CMB. Second, a confirmatory factor analysis was carried out to assess the presence of an unmeasured method factor. Since the results were similar to those obtained earlier when the factor was included, this suggested that there was little evidence for CMB inflating the results (Podsakoff et al., 2012).

## 4 Results

### 4.1 Data screening and respondent profile

#### 4.1.1 Data screening and outlier analysis

After listwise deletion of incomplete responses, the final dataset comprised *N* = 248. Standardized *z*-scores for construct means were computed in Jamovi using MAXABSZ. Applying Hair et al.’s (2019) criterion of |*z*| > 3.29, no univariate outliers were identified, so all 248 cases were retained for subsequent analyses.

#### 4.1.2 Respondent profile

Table 2 summarizes the demographics of the 248 English teachers from a technology-oriented university in Northeast China. Most participants were male (64.5%, *n* = 160), with females comprising 35.5% (*n* = 88). In terms of age, 27.4% (*n* = 68) were 20–30, 52.0% (*n* = 129) were 31–45, and 20.6% (*n* = 51) were 46–55. The sample thus includes a mix of early-, mid-, and later-career teachers, providing a reasonable basis for examining AI-supported teaching continuance.

**Table 2:** Demographic profile of respondents (*N* = 248).

Variable	Category	Counts	% of Total
Gender	Female	88	35.50%
	Male	160	64.50%
Age group	20–30	68	27.40%
	31–45	129	52.00%
	46–55	51	20.60%

#### 4.1.3 Normality assessment

Normality was assessed via skewness and kurtosis statistics for all items (Table 3). Skewness ranged from −0.71 to −0.12 and kurtosis from −0.93 to 0.33, all within acceptable ranges for SEM with maximum likelihood estimation (|skewness| ≤ 2, |kurtosis| ≤ 7; Byrne, 2016; Kline, 2023). The data were therefore suitable for factor analysis and SEM.

**Table 3:** Normality assessment: skewness and kurtosis values for measurement items (*N* = 248).

Item	*N*	Skewness		Kurtosis	
		Skewness	SE	Kurtosis	SE
PU1	248	−0.122	0.155	−0.4764	0.308
PU2	248	−0.454	0.155	−0.6473	0.308
PU3	248	−0.296	0.155	−0.4864	0.308
PU4	248	−0.36	0.155	−0.7387	0.308
PU5	248	−0.337	0.155	−0.9272	0.308
PU6	248	−0.386	0.155	−0.6002	0.308
TTF1	248	−0.586	0.155	0.2175	0.308
TTF2	248	−0.275	0.155	−0.5471	0.308
TTF3	248	−0.444	0.155	−0.4798	0.308
TTF4	248	−0.143	0.155	−0.6107	0.308
TTF5	248	−0.576	0.155	0.035	0.308
TTF6	248	−0.356	0.155	−0.607	0.308
C1	248	−0.213	0.155	−0.6818	0.308
C2	248	−0.532	0.155	−0.422	0.308
C3	248	−0.388	0.155	−0.2822	0.308
C4	248	−0.391	0.155	−0.5768	0.308
PEOU1	248	−0.71	0.155	0.1048	0.308
PEOU2	248	−0.297	0.155	−0.6167	0.308
PEOU3	248	−0.621	0.155	−0.463	0.308
PEOU4	248	−0.64	0.155	−0.1552	0.308
S1	248	−0.493	0.155	−0.1357	0.308
S2	248	−0.486	0.155	0.1232	0.308
S3	248	−0.37	0.155	−0.0405	0.308
S4	248	−0.419	0.155	−0.5738	0.308
S5	248	−0.328	0.155	−0.2834	0.308
S6	248	−0.569	0.155	−0.0333	0.308
BI1	248	−0.119	0.155	−0.7442	0.308
BI2	248	−0.524	0.155	−0.1491	0.308
BI3	248	−0.322	0.155	−0.239	0.308
BI4	248	−0.461	0.155	−0.4483	0.308
BI5	248	−0.16	0.155	−0.8104	0.308
BI6	248	−0.512	0.155	0.3299	0.308
UB1	248	−0.295	0.155	−0.623	0.308
UB2	248	−0.386	0.155	−0.5905	0.308
UB3	248	−0.486	0.155	−0.2469	0.308
UB4	248	−0.293	0.155	−0.6023	0.308
UB5	248	−0.265	0.155	−0.5225	0.308
UB6	248	−0.586	0.155	0.1463	0.308

### 4.2 Descriptive statistics of latent variables

Construct-level descriptive statistics are reported in Table 4. Overall, teachers evaluated the AI assistant positively, with means ranging from 3.91 to 4.04 on a 5-point scale. Perceived Ease of Use (PEOU; *M* = 4.04, SD = 0.74) and Perceived Usefulness (PU; *M* = 4.00, SD = 0.66) were highest, followed closely by Task–Technology Fit (TTF; *M* = 3.99, SD = 0.63) and Use Behavior (UB; *M* = 3.98, SD = 0.65). Satisfaction (S; *M* = 3.96, SD = 0.65), Confirmation (C; *M* = 3.93, SD = 0.72), and Behavioral Intention (BI; *M* = 3.91, SD = 0.70) were also moderately high, indicating favorable post-adoption perceptions.

**Table 4:** Descriptive statistics of latent variables (*N* = 248).

Variable	Mean	SD	Minimum	Maximum
Perceived usefulness (PU)	4	0.658	2.33	5
Task–technology fit (TTF)	3.99	0.628	2	5
Confirmation (C)	3.93	0.716	2	5
Perceived ease of use (PEOU)	4.04	0.741	1.75	5
Satisfaction (S)	3.96	0.649	1.5	5
Behavioral intention (BI)	3.91	0.696	1.67	5
Use behavior (UB)	3.98	0.648	2.17	5

### 4.3 Exploratory factor analysis (EFA)

An EFA was conducted to verify the dimensionality of the adapted items prior to CFA, as recommended for SEM-based research (Fabrigar and Wegener, 2012; Hair et al., 2019). Using minimum residual extraction with oblimin rotation (Costello and Osborne, 2005), a clear seven-factor solution emerged that corresponded to PU, TTF, C, PEOU, S, BI, and UB (Table 5).

**Table 5:** Exploratory factor analysis: factor loadings and uniqueness values.

Item	Factor							Uniqueness
	F1 (BI)	F2 (PU)	F3 (TTF)	F4 (PEOU)	F5 (UB)	F6 (C)	F7 (S)	
PU1		0.731						0.435
PU2		0.72						0.395
PU3		0.73						0.478
PU4		0.811						0.295
PU5		0.828						0.317
PU6		0.766						0.402
TTF1				0.716				0.458
TTF2				0.688				0.447
TTF3				0.576				0.553
TTF4				0.697				0.421
TTF5				0.783				0.406
TTF6				0.792				0.37
C1							0.704	0.484
C2							0.801	0.368
C3							0.796	0.343
C4							0.78	0.288
PEOU1						0.671		0.447
PEOU2						0.838		0.309
PEOU3						0.815		0.355
PEOU4						0.863		0.196
S1					0.563			0.456
S2					0.681			0.458
S3					0.637			0.519
S4					0.707			0.418
S5					0.808			0.336
S6					0.775			0.352
BI1	0.724							0.319
BI2	0.749							0.38
BI3	0.699							0.441
BI4	0.78							0.345
BI5	0.734							0.376
BI6	0.808							0.356
UB1			0.718					0.409
UB2			0.717					0.457
UB3			0.736					0.457
UB4			0.77					0.388
UB5			0.678					0.483
UB6			0.791					0.358

‘Minimum residual’ extraction method was used in combination with a ‘oblimin’ rotation.

All items loaded substantially on their intended factors (loadings > 0.50), with no problematic cross-loadings. Uniqueness values (0.196–0.553) indicated that each factor explained a substantial proportion of variance in its indicators. These results provide strong preliminary evidence for the seven-factor structure and justify proceeding to CFA.

### 4.4 Confirmatory factor analysis (CFA)

CFA was used to evaluate the measurement model’s factorial validity, reliability, and convergent and discriminant validity (Hair et al., 2019; Kline, 2023). Analyses were conducted in Jamovi using the lavaan-based SEM module.

#### 4.4.1 Model fit evaluation

The seven-factor measurement model demonstrated excellent fit (Table 6). RMSEA = 0.036 indicated a close fit to the data, while CFI = 0.960 and TLI = 0.956 exceeded the recommended 0.90 thresholds (Navarro and Foxcroft, 2022; Byrne, 2016; Kline, 2023). Overall, the measurement model provided a sound basis for structural analyses.

**Table 6:** Confirmatory factor analysis model fit indices.

Fit index	Acceptable criteria	Source	Statistical values
RMSEA	≤0.08	Navarro and Foxcroft (2022)	0.0361
CFI	≥0.90	Navarro and Foxcroft (2022)	0.96
TLI	≥0.90	Navarro and Foxcroft (2022)	0.956
Model summary			In harmony with empirical data

#### 4.4.2 Convergent validity and composite reliability

Standardized factor loadings, Average Variance Extracted (AVE), and Composite Reliability (CR) are reported in Table 7. All loadings exceeded 0.50 and were significant at *p* < 0.001, indicating strong item–construct relations.

**Table 7:** Convergent validity and composite reliability of the measurement model.

Factor	Indicator	Estimate	SE	*Z*	*p*	Stand. estimate	AVE (>0.5)	CR (>0.7)	SQRT AVE
Perceived usefulness	PU1	0.647	0.048	13.500	<0.001	0.753	0.600	0.900	0.775
	PU2	0.618	0.044	14.000	<0.001	0.776			
	PU3	0.554	0.045	12.400	<0.001	0.711			
	PU4	0.650	0.042	15.500	<0.001	0.830			
	PU5	0.645	0.043	15.100	<0.001	0.815			
	PU6	0.620	0.046	13.600	<0.001	0.758			
Task–technology fit	TTF1	0.576	0.045	12.800	<0.001	0.732	0.544	0.877	0.738
	TTF2	0.568	0.043	13.100	<0.001	0.745			
	TTF3	0.544	0.049	11.000	<0.001	0.657			
	TTF4	0.630	0.047	13.400	<0.001	0.757			
	TTF5	0.618	0.047	13.100	<0.001	0.745			
	TTF6	0.584	0.042	14.100	<0.001	0.783			
Confirmation	C1	0.606	0.050	12.200	<0.001	0.712	0.617	0.865	0.785
	C2	0.637	0.046	13.900	<0.001	0.781			
	C3	0.672	0.047	14.200	<0.001	0.796			
	C4	0.742	0.048	15.500	<0.001	0.846			
Perceived ease of use	PEOU1	0.611	0.047	13.100	<0.001	0.741	0.662	0.886	0.814
	PEOU2	0.694	0.046	15.200	<0.001	0.820			
	PEOU3	0.647	0.045	14.300	<0.001	0.786			
	PEOU4	0.839	0.048	17.500	<0.001	0.900			
Satisfaction	S1	0.606	0.048	12.600	<0.001	0.724	0.556	0.882	0.746
	S2	0.604	0.047	12.800	<0.001	0.728			
	S3	0.561	0.047	12.000	<0.001	0.696			
	S4	0.564	0.042	13.400	<0.001	0.753			
	S5	0.662	0.047	14.200	<0.001	0.785			
	S6	0.658	0.046	14.200	<0.001	0.785			
Behavioral intention	BI1	0.683	0.044	15.600	<0.001	0.829	0.617	0.906	0.786
	BI2	0.688	0.049	14.100	<0.001	0.776			
	BI3	0.600	0.045	13.300	<0.001	0.744			
	BI4	0.657	0.044	14.900	<0.001	0.803			
	BI5	0.663	0.048	13.800	<0.001	0.764			
	BI6	0.677	0.046	14.600	<0.001	0.795			
Use behavior	UB1	0.665	0.048	13.800	<0.001	0.770	0.563	0.885	0.751
	UB2	0.629	0.049	12.900	<0.001	0.735			
	UB3	0.544	0.044	12.300	<0.001	0.711			
	UB4	0.643	0.046	13.900	<0.001	0.775			
	UB5	0.564	0.046	12.400	<0.001	0.714			
	UB6	0.608	0.042	14.400	<0.001	0.794			

AVE values ranged from 0.544 (TTF) to 0.662 (PEOU), surpassing the 0.50 criterion for convergent validity (Fornell and Larcker, 1981; Hair et al., 2019). PU (AVE = 0.600), C (0.617), S (0.556), BI (0.617), and UB (0.563) also showed satisfactory convergence.

CR values ranged from 0.865 (C) to 0.906 (BI), with TTF (0.877), PEOU (0.886), S (0.882), PU (0.900), and UB (0.885) all above the 0.70 threshold. This indicates excellent internal consistency across all constructs (Hair et al., 2019).

#### 4.4.3 Discriminant validity

Discriminant validity was examined using the Fornell–Larcker criterion. As shown in Table 8, the square roots of AVE (diagonal values: 0.738–0.814) exceeded all inter-construct correlations in the corresponding rows and columns, confirming that each construct was empirically distinct (Fornell and Larcker, 1981). For example, the square root of AVE for PU (0.775) was higher than its correlations with TTF, C, PEOU, S, BI, and UB.

**Table 8:** Fornell–Larcker discriminant validity assessment.

Mean	M-PU	M_TTF	M_C	M_PEOU	M_S	M_BI	M_UB
M-PU	**0.775**						
M_TTF	0.112	**0.738**					
M_C	0.269	0.236	**0.785**				
M_PEOU	0.156	0.257	0.230	**0.814**			
M_S	0.340	0.373	0.391	0.362	**0.746**		
M_BI	0.288	0.420	0.318	0.395	0.436	**0.786**	
M_UB	0.230	0.193	0.301	0.284	0.366	0.431	**0.751**

Bold values on the diagonal represent the square root of the Average Variance Extracted (AVE); off-diagonal values are inter-construct correlations (Fornell and Larcker, 1981).

#### 4.4.4 Summary

Taken together, the EFA and CFA results indicate that the measurement model is psychometrically robust. The seven-factor structure shows excellent fit, strong convergent validity (high, significant loadings and AVE ≥ 0.50), high composite reliability (CR ≥ 0.86), and clear discriminant validity (Fornell–Larcker). This provides a solid foundation for testing the structural relationships among TTF, PEOU, PU, C, S, BI, and UB.

### 4.5 Structural equation modeling (SEM)

**Figure 4:**
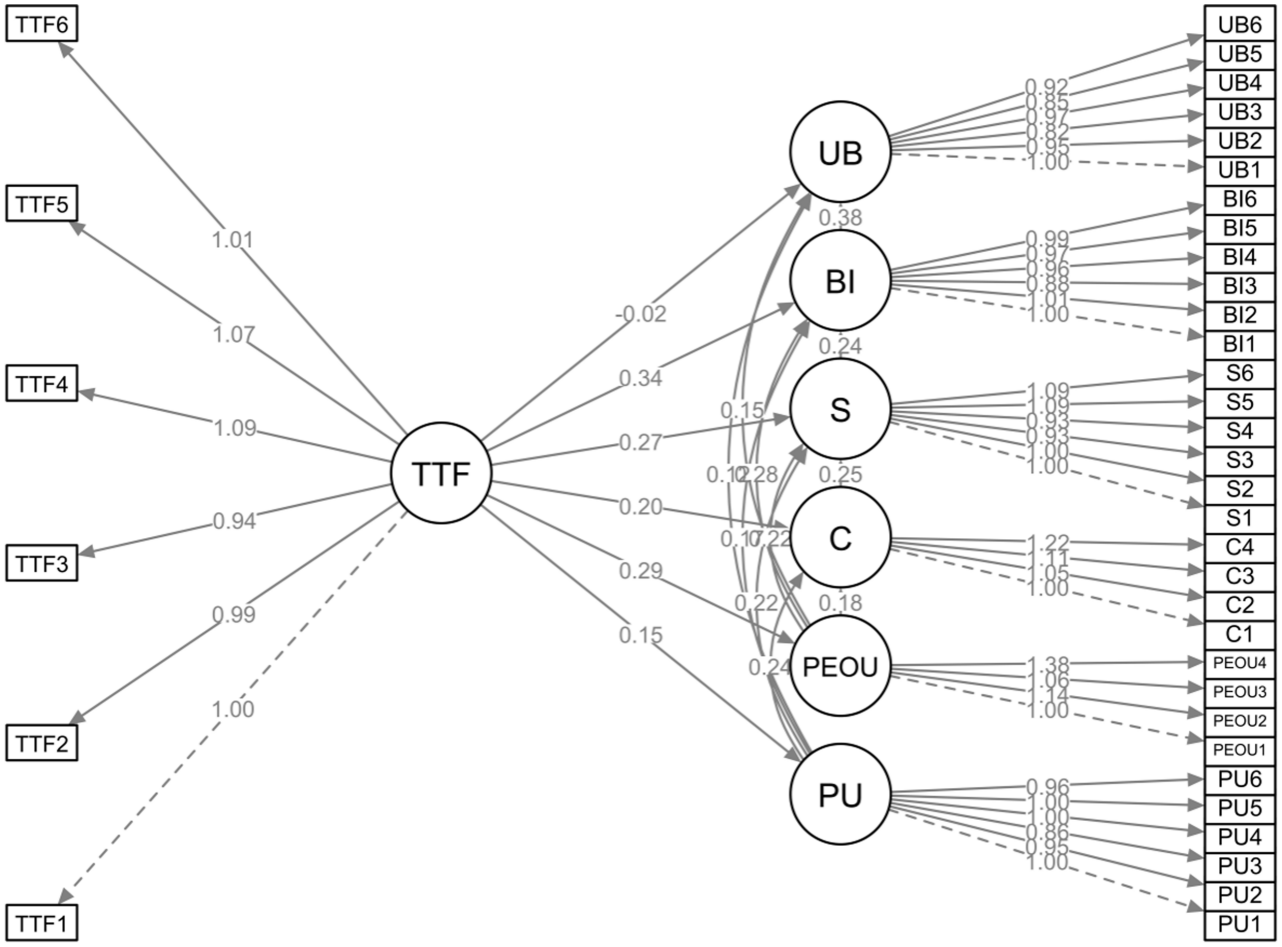
The Structural Equation Model (SEM) path diagram.

SEM was used to assess the hypothesized relationships within the integrated TTF–TAM–ECM framework (Hair et al., 2019; Kline, 2023). Figure 4 displays the tested model.

#### 4.5.1 Structural model fit

The structural model exhibited excellent fit (Table 9). GFI = 0.976, CFI = 0.957, and TLI = 0.954 exceeded recommended minima (≥0.80 or ≥0.90, depending on the index), and RMSEA = 0.037 and SRMR = 0.056 were below widely accepted upper bounds (Cho et al., 2020; Hoope et al., 2008; Sharma et al., 2005). The structural specification is therefore consistent with the observed data.

**Table 9:** Fit indices results of the structural equation model.

Fit index	Acceptable criteria	Source	Statistical values
GFI	≥0.80	Cho et al.	0.976
SRMR	≤0.08	Cho et al.	0.056
RMSEA	≤0.10	Hooper et al.	0.037
CFI	≥0.80	Hooper et al.	0.957
TLI	≥0.80	Sharma et al.	0.954
Model summary			In harmony with empirical data

#### 4.5.2 Path coefficients

Table 10 summarizes all direct effects. Standardized coefficients (*β*), *z*-values, *p*-values, and confidence intervals were used to evaluate support for each hypothesized path (Hair et al., 2019; Kline, 2023).

**Table 10:** Path coefficients.

Dep	Pred	Estimate	SE	95% Confidence Intervals		*β*	*z*	*p*
				Lower	Upper			
PU	TTF	0.1496	0.0803	−0.00784	0.307	0.1332	1.862	0.063
PEOU	TTF	0.2947	0.0772	0.14338	0.446	0.2782	3.817	<0.001
C	PU	0.2412	0.066	0.11185	0.371	0.2585	3.655	<0.001
C	PEOU	0.1781	0.0709	0.03917	0.317	0.18	2.512	0.012
C	TTF	0.1992	0.077	0.04834	0.35	0.19	2.588	0.01
S	PU	0.2196	0.0603	0.10141	0.338	0.2373	3.642	<0.001
S	PEOU	0.2237	0.0646	0.09705	0.35	0.2279	3.462	<0.001
S	C	0.2506	0.0701	0.11332	0.388	0.2526	3.577	<0.001
S	TTF	0.2709	0.071	0.13183	0.41	0.2606	3.817	<0.001
BI	PU	0.1699	0.0669	0.03883	0.301	0.1624	2.541	0.011
BI	PEOU	0.2783	0.0739	0.13352	0.423	0.2508	3.768	<0.001
BI	S	0.2384	0.0865	0.06887	0.408	0.2109	2.756	0.006
BI	TTF	0.3427	0.0822	0.18166	0.504	0.2916	4.171	<0.001
UB	PU	0.1245	0.0687	−0.01017	0.259	0.1221	1.812	0.07
UB	PEOU	0.147	0.0782	−0.00625	0.3	0.1361	1.88	0.06
UB	BI	0.3849	0.0846	0.21913	0.551	0.3952	4.551	<0.001
UB	TTF	−0.0181	0.0859	−0.18651	0.15	−0.0158	−0.211	0.833

Key findings are summarized below.

##### 4.5.2.1 Effects of TTF on PU, PEOU, C, S, BI, and UB (H1–H6)

TTF significantly predicted PEOU (H2; *β* = 0.278, *p* < 0.001), C (H3; *β* = 0.190, *p* = 0.010), S (H4; *β* = 0.261, *p* < 0.001), and BI (H5; *β* = 0.292, *p* < 0.001), but not PU (H1; *β* = 0.133, *p* = 0.063) or UB (H6; *β* = −0.016, *p* = 0.833). Thus, better task–technology alignment enhanced ease of use, confirmation, satisfaction, and intention, but did not directly translate into perceived usefulness or actual use.

##### 4.5.2.2 Effects of PU and PEOU on confirmation and satisfaction (H7–H11)

Both PU and PEOU positively influenced C (H7: *β* = 0.259, *p* < 0.001; H8: *β* = 0.180, *p* = 0.012), consistent with ECM’s emphasis on post-use cognitive evaluations (Bhattacherjee, 2001). PU (H9; *β* = 0.237, *p* < 0.001), PEOU (H10; *β* = 0.228, *p* < 0.001), and C (H11; *β* = 0.253, *p* < 0.001) all significantly predicted S, indicating that satisfaction is jointly shaped by usefulness beliefs, ease perceptions, and confirmation experiences.

##### 4.5.2.3 Effects on behavioral intention (H12, H14, H15)

BI was significantly predicted by S (H12; *β* = 0.211, *p* = 0.006), PU (H14; *β* = 0.162, *p* = 0.011), PEOU (H15; *β* = 0.251, *p* < 0.001), and TTF (H5; *β* = 0.292, *p* < 0.001). This pattern highlights the combined contribution of cognitive evaluations (PU, PEOU), affective responses (S), and task alignment (TTF) to intention.

##### 4.5.2.4 Effects on use behavior (H13, H16, H17)

UB was driven primarily by BI (H13; *β* = 0.395, *p* < 0.001). Direct effects of PU (H16; *β* = 0.122, *p* = 0.070), PEOU (H17; *β* = 0.136, *p* = 0.060), and TTF (H6; *β* = −0.016, *p* = 0.833) on UB were not significant. This pattern aligns with behavioral models such as TAM and UTAUT, where intention mediates the influence of beliefs on actual behavior (Davis, 1989; Venkatesh et al., 2003).

Overall, TTF, PU, PEOU, and C jointly shape S and BI, while UB is largely intention-driven.

#### 4.5.3 Mediation analysis

To unpack the mechanisms linking key constructs, mediation analysis was conducted using bootstrapped indirect effects with 95% bias-corrected confidence intervals (Baron and Kenny, 1986; Shrout and Bolger, 2002; Hayes and Rockwood, 2020; Zhao et al., 2010). Indirect effects are summarized in Table 11; mediation is supported when the CI does not include zero.

**Table 11:** Indirect effects for mediation analysis.

Label	Description	Parameter	Estimate	SE	95% Confidence Intervals		β	z	p
					Lower	Upper			
IE1	PU ⇒ C ⇒ S	p17*p18	0.104	0.031	0.043	0.166	0.114	3.347	<0.001
IE2	PU ⇒ S ⇒ BI	p19*p20	0.169	0.042	0.087	0.252	0.162	4.032	<0.001
IE3	PU ⇒ BI ⇒ UB	p19*p21	0.146	0.039	0.069	0.222	0.142	3.718	<0.001
IE4	PEOU ⇒ C ⇒ S	p15*p17	0.098	0.032	0.036	0.16	0.099	3.093	0.002
IE5	PEOU ⇒ S ⇒ BI	p17*p19	0.164	0.042	0.082	0.246	0.147	3.919	<0.001
IE6	PEOU ⇒ BI ⇒ UB	p17*p19	0.202	0.048	0.108	0.295	0.186	4.232	<0.001
IE7	TTF ⇒ PU ⇒ C	p17*p19	0.037	0.023	−0.007	0.082	0.035	1.65	0.099
IE8	TTF ⇒ PU ⇒ S	p19*p21	0.045	0.026	−0.006	0.095	0.043	1.727	0.084
IE9	TTF ⇒ PU ⇒ BI	p19*p21	0.04	0.024	−0.007	0.086	0.033	1.68	0.093
IE10	TTF ⇒ PU ⇒ UB	p19*p21	0.035	0.022	−0.008	0.077	0.03	1.6	0.11
IE11	TTF ⇒ PEOU ⇒ UB	p19*p18	0.088	0.032	0.026	0.151	0.077	2.76	0.006
IE12	TTF ⇒ PEOU ⇒ BI	p17*p19	0.11	0.035	0.042	0.178	0.093	3.185	0.001
IE13	TTF ⇒ PEOU ⇒ S	p17*p19	0.089	0.03	0.031	0.148	0.085	2.997	0.003
IE14	TTF ⇒ PEOU ⇒ C	p15*p17	0.061	0.026	0.01	0.112	0.058	2.328	0.02

##### 4.5.3.1 Mediation effects involving PU and PEOU (H18–H23)

Confirmation significantly mediated the effects of PU and PEOU on S (IE1: *β* = 0.114, *p* < 0.001; IE4: *β* = 0.099, *p* = 0.002), supporting H18 and H19. S, in turn, mediated the effects of PU and PEOU on BI (IE2: *β* = 0.162, *p* < 0.001; IE5: *β* = 0.147, *p* < 0.001), supporting H20 and H21. BI mediated the effects of PU and PEOU on UB (IE3: *β* = 0.142, *p* < 0.001; IE6: *β* = 0.186, *p* < 0.001), supporting H22 and H23. These results underscore a multi-step mechanism: usefulness and ease perceptions → confirmation and satisfaction → intention → actual use.

##### 4.5.3.2 Mediation effects involving TTF through PU (H24–H27)

Indirect effects from TTF through PU to C, S, BI, and UB (IE7–IE10) were not significant (CIs included zero), so H24–H27 were not supported. TTF’s influence does not appear to operate primarily through usefulness.

##### 4.5.3.3 Mediation effects involving TTF through PEOU (H28–H31)

In contrast, TTF exerted significant indirect effects via PEOU. PEOU mediated the effects of TTF on C (IE14: *β* = 0.058, *p* = 0.020), S (IE13: *β* = 0.085, *p* = 0.003), BI (IE12: *β* = 0.093, *p* = 0.001), and UB (IE11: *β* = 0.077, *p* = 0.006), supporting H28–H31. This highlights usability as a key channel through which task–technology alignment shapes confirmation, satisfaction, intention, and actual use in AI-supported teaching.

#### 4.5.4 Summary of hypothesis testing

Table 12 summarizes all 31 hypotheses. In total, 25 hypotheses were supported and 6 were not.

- TTF significantly enhanced PEOU, C, S, and BI, but not PU or UB.
- PU and PEOU significantly influenced C, S, and BI, but not UB directly.
- S significantly predicted BI, and BI strongly predicted UB.
- Indirectly, PU and PEOU affected S via C, BI via S, and UB via BI.
- TTF exerted robust indirect effects via PEOU (but not via PU) on C, S, BI, and UB.

**Table 12:** Summary of hypothesis testing.

Hypothesis	Standardized Coefficients (*β*)	*z*-value	*p*	Result
H1: Task–Technology Fit (TTF) positively influences Perceived Usefulness (PU).	0.133	1.862	0.063	Not supported
H2: Task–Technology Fit (TTF) positively influences Perceived Ease of Use (PEOU).	0.278	3.817	<0.001	Supported
H3: Task–Technology Fit (TTF) positively influences Confirmation.	0.19	2.588	0.01	Supported
H4: Task–Technology Fit (TTF) positively influences Satisfaction.	0.261	3.817	<0.001	Supported
H5: Task–Technology Fit (TTF) positively influences Behavioral Intention.	0.292	4.171	<0.001	Supported
H6: Task–Technology Fit (TTF) positively influences Use Behavior.	−0.016	−0.211	0.833	Not supported
H7: Perceived Usefulness (PU) positively influences Confirmation.	0.259	3.655	<0.001	Supported
H8: Perceived Ease of Use (PEOU) positively influences Confirmation.	0.18	2.512	0.012	Supported
H9: Perceived Usefulness (PU) positively influences Satisfaction.	0.237	3.642	<0.001	Supported
H10: Perceived Ease of Use (PEOU) positively influences Satisfaction.	0.228	3.462	<0.001	Supported
H11: Confirmation positively influences Satisfaction.	0.253	3.577	<0.001	Supported
H12: Satisfaction positively influences Behavioral Intention.	0.211	2.756	0.006	Supported
H13: Continuance Intention positively influences Use Behavior.	0.395	4.551	<0.001	Supported
H14: Perceived Usefulness (PU) positively influences Behavioral Intention.	0.162	2.541	0.011	Supported
H15: Perceived Ease of Use (PEOU) positively influences Behavioral Intention.	0.251	3.768	<0.001	Supported
H16: Perceived Usefulness (PU) positively influences Use Behavior.	0.122	1.812	0.07	Not supported
H17: Perceived Ease of Use (PEOU) positively influences Use Behavior.	0.136	1.88	0.06	Not supported
H18: The effect of PU on Satisfaction is mediated by Confirmation.	0.114	3.347	<0.001	Supported
H19: The effect of PEOU on Satisfaction is mediated by Confirmation.	0.099	3.093	0.002	Supported
H20: The effect of PU on Behavioral Intention is mediated by Satisfaction.	0.162	4.032	<0.001	Supported
H21: The effect of PEOU on Behavioral Intention is mediated by Satisfaction.	0.147	3.919	<0.001	Supported
H22: The effect of PU on Use Behavior is mediated by Behavioral Intention.	0.142	3.718	<0.001	Supported
H23: The effect of PEOU on Use Behavior is mediated by Behavioral Intention.	0.186	4.232	<0.001	Supported
H24: The effect of TTF on Confirmation is mediated by Perceived Usefulness.	0.035	1.65	0.099	Not supported
H25: The effect of TTF on Satisfaction is mediated by Perceived Usefulness.	0.043	1.727	0.084	Not supported
H26: The effect of TTF on Behavioral Intention is mediated by Perceived Usefulness.	0.033	1.68	0.093	Not supported
H27: The effect of TTF on Use Behavior is mediated by Perceived Usefulness.	0.03	1.6	0.11	Not supported
H28: The effect of TTF on Confirmation is mediated by Perceived Ease of Use.	0.058	2.328	0.02	Supported
H29: The effect of TTF on Satisfaction is mediated by Perceived Ease of Use.	0.085	2.997	0.003	Supported
H30: The effect of TTF on Behavioral Intention is mediated by Perceived Ease of Use.	0.093	3.185	0.001	Supported
H31: The effect of TTF on Use Behavior is mediated by Perceived Ease of Use.	0.077	2.76	0.006	Supported

These findings provide strong empirical support for the integrated TTF–TAM–ECM model and underscore the central roles of PEOU, satisfaction, and behavioral intention in sustaining teachers’ use of an AI teaching assistant in higher education.

## 5 Discussion

### 5.1 Main findings

This study investigated university English teachers’ post-adoption perceptions and continuance use of the Superstar AI Assistant using an integrated TTF–TAM–ECM model. After 4 months of daily use, teachers reported generally positive evaluations of the system, and the structural model revealed a coherent chain from task–technology alignment and cognitive appraisals to satisfaction, behavioral intention, and actual use. Perceived Usefulness (PU), Perceived Ease of Use (PEOU), Confirmation, Satisfaction, and Task–Technology Fit (TTF) each played distinct, complementary roles.

Behavioral Intention emerged as the only direct predictor of Use Behavior (*β* = 0.395, *p* < 0.001), consistent with post-adoption technology literature emphasizing the intention–behavior link in educational contexts (Wang et al., 2025; Xue et al., 2025). Neither PU nor PEOU directly predicted actual use, suggesting that once AI systems are routinized, continued engagement is driven less by isolated beliefs about usefulness or usability and more by a consolidated intention to keep using the tool, echoing recent findings on “habitualized” AI teaching (Hazzan-Bishara et al., 2025; Or, 2025).

TTF showed strong effects on PEOU, Confirmation, Satisfaction, and Behavioral Intention, but not on PU or Use Behavior. Teachers clearly perceived that the AI assistant aligned with core tasks (e.g., lesson planning, grading, analytics), and this alignment enhanced ease of use, affective evaluation, and intention, but did not automatically translate into perceived performance gains. This reinforces arguments that AI in teaching often functions as a workload-reduction and coordination tool rather than a straightforward pedagogical “upgrade” (Ifenthaler and Yau, 2020; Lund and Wang, 2023).

Confirmation and Satisfaction played central mediating roles. PU and PEOU significantly predicted Confirmation, which in turn strengthened Satisfaction, and Satisfaction mediated key links from PU and PEOU to Behavioral Intention, in line with ECM (Bhattacherjee, 2001) and emerging AI-in-education findings on the emotional basis of continuance (Zhang, 2025; Baig and Yadegaridehkordi, 2025). Significant chains such as PU → C → S, PU → S → BI, PEOU → S → BI, and BI → UB indicate that continuance is driven by interlocking cognitive and affective processes rather than any single variable (Or, 2025).

Overall, sustained use of the Superstar AI Assistant appears to arise from task alignment plus positive experiential confirmation, which feed Satisfaction and Behavioral Intention and culminate in stable, intention-driven usage patterns.

### 5.2 Interpretation of key relationships

The structural model confirms that even in a post-adoption phase, PU and PEOU remain important for shaping Confirmation and Satisfaction, indicating that teachers continue to monitor effort and effectiveness as they work with AI systems (Kim, 2024; Huang et al., 2023). Ease of interaction and perceived functional benefits thus remain salient beyond initial adoption.

TTF exerted consistent influence on PEOU, Confirmation, Satisfaction, and Behavioral Intention, revealing that contextual alignment between AI features and teachers’ actual tasks is a key driver of ongoing engagement. When teachers perceive that AI-supported functions (e.g., automated feedback, resource recommendation, student monitoring) map onto their real instructional responsibilities, they report greater experiential confirmation and affective satisfaction, aligning with research that foregrounds ecological fit in AI-supported teaching (Ifenthaler and Yau, 2020; Hazzan-Bishara et al., 2025).

However, TTF did not significantly predict PU, diverging from traditional TTF assumptions where good fit typically enhances perceived performance (Goodhue and Thompson, 1995). In AI teaching assistants, teachers may differentiate between “doing tasks more smoothly” and “genuinely improving pedagogy.” Recent work suggests that while AI can substantially reduce workload, teachers remain cautious about attributing deeper pedagogical value to AI—especially in disciplines that rely heavily on nuanced, human judgment such as language teaching (Lund and Wang, 2023; Lee and Moore, 2024). The present findings add nuance by showing that fit may matter more for experiential and affective responses than for perceived performance enhancement.

Satisfaction emerged as a powerful predictor of Behavioral Intention and a central mediator for upstream constructs, reinforcing its status as a “psychological anchor” in post-adoption contexts (Bhattacherjee, 2001; Hazzan-Bishara et al., 2025; Baig and Yadegaridehkordi, 2025). Satisfaction appears to synthesize multiple signals—ease, fit, confirmation—into a durable motivational state that sustains engagement. Finally, the robust intention–behavior link, coupled with non-significant direct paths from PU, PEOU, and TTF to Use Behavior, suggests a shift from evaluation-driven to intention-driven and routinized use, consistent with arguments that AI adoption in teaching becomes gradually embedded in everyday workflows (Xue et al., 2025; Or, 2025).

Taken together, the interplay among TTF, PU, PEOU, Confirmation, and Satisfaction supports the value of integrating TAM, ECM, and TTF, while also pointing to AI-specific patterns—particularly the centrality of task alignment and affective consolidation in sustaining long-term teacher–AI collaboration.

### 5.3 Theoretical interpretation of non-significant direct paths

The non-significant direct effects of TTF on PU (H3) and actual use behavior deserve particular theoretical consideration. The non-significance of the direct effect of TTF on PU and actual use behavior does not imply that TTF is an insignificant factor in technology adoption contexts but that TTF is a distal factor whose influence on subsequent outcomes is mediated by affective and cognitive outcomes such as satisfaction and perceived ease of use.

Three potential explanations exist to account for our findings. Firstly, our findings are consistent with the mediation-dominant models of TTF proposed in prior TTF research (Larsen et al., 2009; Wu and Chen, 2017), where the effect of TTF on behavioral outcomes is mediated by intervening outcomes. Our mediation analyses provided evidence of significant mediated relationships between TTF and behavioral intention/use through satisfaction, implying that the effect of TTF on behavioral intention/use is fully mediated by satisfaction. When users experience high levels of task-technology fit, they report high levels of satisfaction, which in turn influence continuance behavior.

Second, TTF and PU may be measuring conceptually proximate but nevertheless distinct dimensions of evaluation. TTF measures a judgment about the functional appropriateness of the fit between task and system, whereas PU measures a more general evaluation of the benefits to productivity. In a situation where the AI assistant is already judged to be highly appropriate to instructional tasks, teachers may experience ease of use and satisfaction effects before attributing benefits to the system. This may reduce the relationship between TTF and PU.

Third, contextual saturation effects may be at work. In a situation where the adoption of AI assistants has become institutionalized throughout the instructional process, teachers may take the task fit for granted. In such a situation, the effects of confirmation and satisfaction may be the immediate determinants of continued use, whereas the functional fit recedes into the background (Bhattacherjee, 2001; Dishaw and Strong, 1999).

Other factors may be at work. Perhaps the non-significant relationships between TTF and PU are partly explained by the specificity of the measures. TTF items measured teachers’ perceptions about the system in specific task domains, whereas PU items measured teachers’ perceptions about the system in general. Perhaps the range of TTF was constrained by the fact that the teachers in the study were experienced and voluntarily using AI assistants.

### 5.4 Theoretical contributions

This study makes several contributions to technology-continuance theory in AI-supported higher education. First, it highlights TTF as a central driver in AI-based teaching contexts. Whereas earlier work often treated TTF as a complementary antecedent to PU (Goodhue and Thompson, 1995), here TTF strongly predicts PEOU, Confirmation, Satisfaction, and Behavioral Intention without directly shaping PU. This suggests that in AI-mediated teaching, perceived fit between AI capabilities and pedagogical tasks may exert more influence via experiential and affective pathways than via classical performance expectations, echoing recent calls to recalibrate technology theories for AI-enhanced contexts (Hazzan-Bishara et al., 2025; Ifenthaler and Yau, 2020).

Second, the findings support conceptualizing AI not as a static tool but as a socio-cognitive partner with whom teachers develop evolving relationships. The persistent influence of PEOU and Confirmation on Satisfaction indicates that teachers’ judgments are formed iteratively as they accumulate interaction experience, rather than at a single acceptance moment. This aligns with emerging work that frames AI adoption as cyclical learning and reflection (Kim, 2024; Cosgun, 2025). The study thus reinforces the need to move from one-shot acceptance models toward post-adoptive cognition frameworks that foreground dynamic human–AI interaction.

Third, the validated measurement model contributes a context-sensitive instrument for examining AI teaching assistant adoption in higher education—particularly in language teaching. It provides psychometric support for adapting TAM, ECM, and TTF to AI-specific features, responding to calls for updated measurement approaches in AI-related educational research (Lund and Wang, 2023).

Finally, the study reaffirms Satisfaction as a core mediator linking cognitive evaluations (PU, PEOU, Confirmation) to motivational outcomes and behavior, in line with ECM (Bhattacherjee, 2001) and recent work on motivational and emotional dimensions of AI adoption (Baig and Yadegaridehkordi, 2025). Even with advanced, semi-autonomous systems, human affect remains fundamental to long-term technology integration.

### 5.5 Practical implications

#### 5.5.1 Implications for teachers

For teachers, the strong effects of TTF suggest that AI assistants are most beneficial when deliberately aligned with specific instructional tasks. Rather than using AI in a generic way, teachers may gain more by focusing on features that directly support their own pedagogical goals—such as automating repetitive feedback, structuring lesson plans, or monitoring student progress (Hazzan-Bishara et al., 2025; Lund and Wang, 2023). Given the central role of Satisfaction, iterative experimentation—gradually expanding AI use beyond initial functions—may help teachers discover high-fit, high-value use cases over time (Kim, 2024).

#### 5.5.2 Implications for institutional AI policy

The results have significant implications for institutional AI governance. First, the salience of task-technology fit in the study suggests that institutional AI governance should place greater emphasis on the selection and use of AI systems that offer task functionalities that match teachers’ actual needs in instructional tasks, such as lesson planning, formative assessment, and student feedback, and other aspects of curation.

The importance of confirmation and satisfaction in the study indicates that institutional AI governance should manage teachers’ expectations of AI system performance more effectively, avoiding overpromising and underdelivering, and instead, allowing a phased approach in introducing AI systems where teachers can experience and become satisfied with AI system performance.

The indirect effects of task-technology fit on satisfaction suggest that institutional AI governance should consider the importance of a continuous system of feedback and response to teachers’ concerns, allowing a continued positive experience in AI system use, which in turn would enhance satisfaction and continuance intention.

#### 5.5.3 Implications for teacher professional development

Professional development opportunities should extend beyond technical skills to include these reflective skills in AI integration. Workshops on how to critically evaluate outputs generated by AI systems, determine the best application scenarios, and incorporate AI tools within existing teaching methodologies could be beneficial in creating both perceived usefulness and ease of use.

Another avenue could be peer learning communities where teachers can share their successful experiences in integrating AI systems within their departmental culture. This could be beneficial in creating a collective efficacy among teachers to adopt AI systems within their teaching methodologies.

Finally, professional development opportunities should also be tailored to cover the affective side of AI integration. As discussed in this study, satisfaction is a key driver in creating continuance in user engagement. Workshops or opportunities where teachers can realize and express their benefits from using AI systems, as well as vent their frustrations, could be beneficial in creating a positive post-adoption affect.

#### 5.5.4 Implications for AI system designers

For developers, the study highlights three design priorities: task alignment, usability, and transparency. The strong role of PEOU indicates that intuitive interfaces, clear workflows, and stable performance remain critical, even as AI systems become more complex. In addition, explainable design—providing accessible rationales for recommendations and feedback—may bolster trust and cognitive alignment (Lee and Moore, 2024). Personalization features that adapt to course type, student profile, and teaching style can further enhance TTF and encourage deeper integration.

#### 5.5.5 Implications for policy and educational leadership

For policymakers and educational leaders, the results support institution-wide strategies that frame AI as part of a broader pedagogical ecosystem. Effective AI integration requires not only infrastructure but also clear guidelines for ethical, transparent, and student-centered use; sustained investment in AI-focused professional learning; and robust data protection and accountability frameworks (Ifenthaler and Yau, 2020; Or, 2025). Policies that prioritize teacher experience—by supporting satisfaction, agency, and manageable workload—are more likely to foster durable AI use than policies focused solely on tool deployment (Xue et al., 2025).

#### 5.5.6 Implications for future AI-enhanced learning ecosystems

The dominance of Behavioral Intention in predicting Use Behavior suggests that future AI-enhanced learning ecosystems should be designed to strengthen teachers’ sense of agency and ownership. AI should support, rather than supplant, teacher decision-making by providing timely, interpretable analytics and flexible, teacher-controlled automation. This aligns with emerging models of hybrid human–AI pedagogy, where AI functions as a collaborator that expands human capacity rather than a replacement (Xue et al., 2025; Lund and Wang, 2023).

### 5.6 Integration of supplementary qualitative reflections

Although the study was quantitative-dominant, the optional open-ended reflections provided useful contextualization. The following qualitative excerpts are drawn from teachers’ optional written reflections (*n* = 248) and are presented to contextualize—rather than formally analyze—the quantitative findings. Responses were reviewed descriptively to identify observations resonant with the model’s core constructs; representative quotes were selected for their clarity and relevance to task–technology fit, confirmation, satisfaction, and continuance. These reflections humanize the structural results by illustrating how abstract constructs are experienced in concrete classroom practice.

Many teachers emphasized workload reduction, especially in lesson preparation and assignment evaluation, describing the AI assistant as “saving a tremendous amount of time,” which mirrors the strong TTF–Satisfaction–Intention pathways and aligns with recent findings that day-to-day efficiency is a key driver of AI value (Hazzan-Bishara et al., 2025; Lund and Wang, 2023).

Others highlighted perceived improvements in instructional quality, noting that AI-generated outlines, feedback suggestions, and teaching materials helped them “better support students” and “organize lessons more effectively,” resonating with the quantitative importance of PU. Comments describing the system as “intuitive,” “easy to navigate,” or “stable” reinforced the significance of PEOU, and some explicitly attributed successful adoption to university-provided workshops and technical support, in line with work on institutional facilitation of AI integration (Ifenthaler and Yau, 2020).

A minority of teachers mentioned inconsistent or overly generic AI suggestions, which helps explain why not all direct paths to Use Behavior were significant and why Confirmation was not uniformly high. Overall, these reflections humanize the quantitative results, illustrating how constructs such as TTF, PU, PEOU, Confirmation, and Satisfaction are experienced in concrete classroom practice.

### 5.7 Limitations and future research

Several limitations should be acknowledged. First, the cross-sectional design captures teachers’ perceptions at a single point in time. AI integration is a dynamic process, and constructs such as Confirmation, Satisfaction, and continuance behavior likely evolve as teachers gain experience and as systems are updated (Hazzan-Bishara et al., 2025; Or, 2025). Longitudinal or repeated-measures designs would allow researchers to trace how post-adoption cognition and behavior change over multiple semesters. Moreover, the reliance on self-reported data collected at a single time point raises the possibility of common method bias. Although procedural and statistical remedies were employed (see Section 3.7), future studies using behavioral log data, multi-source ratings, or temporally separated measurements would provide stronger evidence against CMB-driven inflation of structural relationships (Podsakoff et al., 2003).

Second, the sample was drawn from one provincial university in Northeast China. While this enhances ecological validity by situating the study in a real, institution-wide AI implementation, institutional culture, policy environment, and digital infrastructure may differ across contexts (Ifenthaler and Yau, 2020). We have strengthened our acknowledgment of common method bias (CMB) and contextual limitations. Replication in other universities and countries would help test the model’s generalizability and identify context-specific variations.

Third, Use Behavior was measured through self-report, which may be subject to recall or social desirability bias. Integrating system log data—such as frequency and depth of AI-assisted lesson planning, grading, or analytics use—would provide more objective indicators of engagement (Lai and Bower, 2019). Future studies could combine log data with survey measures to examine discrepancies between perceived and actual use.

Fourth, the qualitative component was limited to brief, optional reflections without formal coding. Richer qualitative methods—such as interviews, think-aloud protocols, or classroom observations—could provide deeper insights into how teachers interpret AI-generated suggestions, negotiate errors, and incorporate AI into complex teaching episodes.

Fifth, the model did not explicitly incorporate constructs related to ethics, trust, autonomy, or algorithmic opacity, which are increasingly salient in AI adoption (Lee and Moore, 2024). Exploring how concerns about fairness, transparency, or professional identity intersect with TTF, PU, PEOU, and Satisfaction would deepen understanding of teachers’ ambivalent or critical stances toward AI.

Finally, potential moderators—such as digital literacy, teaching experience, departmental culture, or perceived institutional support—were not examined. Future research could test multi-group or moderated SEM models to explore whether structural relationships differ across teacher subgroups or institutional conditions.

Addressing these limitations through longitudinal, multi-site, log-enhanced, and mixed-methods designs will be crucial for building a more comprehensive account of teachers’ long-term engagement with AI instructional assistants in higher education.

## 6 Conclusion

This study investigated university English teachers’ adoption and continuance use of the Superstar AI Assistant, drawing on TAM, ECM, and TTF and tested via SEM. The findings clarify how cognitive, affective, and task-related mechanisms jointly shape sustained engagement with AI-supported teaching tools.

Task–Technology Fit emerged as a foundational driver, exerting strong effects on Perceived Ease of Use, Confirmation, Satisfaction, and Behavioral Intention. Perceived Usefulness and Perceived Ease of Use further contributed to post-adoption evaluations, while Behavioral Intention was the sole direct predictor of actual Use Behavior, consistent with technology-continuance theory. Mediation analyses showed that Confirmation, Satisfaction, and Behavioral Intention function as key cognitive–affective bridges between system perceptions and sustained use.

Situated in a real institutional context, where teachers used the AI assistant across the full teaching cycle for a semester, the study demonstrates that meaningful AI integration depends not only on usefulness and usability but also on genuine alignment with teachers’ professional tasks and workload realities. The integrated TTF–TAM–ECM framework offers a theoretically grounded lens for informing AI system design, teacher training, and institutional support. Future longitudinal and multisite research can build on this foundation to trace how teacher–AI relationships evolve over time and across diverse higher education settings.

## Data Availability

The original contributions presented in the study are included in the article/Supplementary material, further inquiries can be directed to the corresponding author.
