# Supplementary material for: Understanding university teachers’ continuance of an AI teaching assistant: an integrated TTF–TAM–ECM model in higher education
Source: Front Psychol. 2026 Mar 16;17:1765263. doi: 10.3389/fpsyg.2026.1765263 (PMC13033745; doi:10.3389/fpsyg.2026.1765263)
Supplement: Supplementary file 2 [file Table_2.docx]

Appendix A: Operationalization of Constructs

| **Variable** | **Definition** | **Questionnaire Items** | **Item source** | **Theoretical origin** |
| --- | --- | --- | --- | --- |
| **Task–Technology Fit (TTF)** (6 items) | The degree to which a technology’s capabilities match the requirements of a user’s tasks. | TTF1 Superstar AI Assistant’s functions match my teaching tasks.  TTF2 It provides the features I need for lesson preparation.  TTF3 It supports the teaching and assessment tasks required in my courses.  TTF4 Its outputs are useful for my teaching decisions.  TTF5 It provides relevant information when needed.  TTF6 It integrates well with my existing teaching tools. | Goodhue & Thompson (1995); Dishaw & Strong (1999) | **TTF theory** |
| **Perceived Usefulness (PU)** (6 items) | The extent to which using a system enhances job performance. | PU1 Using Superstar AI Assistant improves my teaching performance.  PU2 It increases my teaching effectiveness.  PU3 It helps me accomplish teaching tasks more quickly. PU4 It enhances my productivity in course preparation.  PU5 It is useful for my English teaching.  PU6 It helps my students achieve better learning outcomes. | Davis (1989); Venkatesh & Davis (2000) | **TAM / UTAUT (Performance Expectancy)** |
| **Perceived Ease of Use (PEOU)** (4 items) | The degree to which using a system is free of effort. | PEOU1 Learning to operate Superstar AI Assistant is easy for me.  PEOU2 Interacting with Superstar AI Assistant is clear and understandable.  PEOU3 I find it easy to become skillful at using Superstar AI Assistant.  PEOU4 Overall, Superstar AI Assistant is easy to use. | Davis (1989) | **TAM / UTAUT (Effort Expectancy)** |
| **Confirmation** (4 items) | The extent to which users’ post-adoption perceptions are consistent with their expectations. | C1 My experience with Superstar AI Assistant was better than I expected.  C2 Overall, Superstar AI Assistant met my expectations.  C3 The performance of Superstar AI Assistant was as I expected.  C4 The benefits I obtained from Superstar AI Assistant were close to what I expected. | Bhattacherjee (2001) | **ECM** |
| **Satisfaction** (6 items) | A positive affective response resulting from prior use of the system. | S1 I am satisfied with my overall experience using Superstar AI Assistant.  S2 Using Superstar AI Assistant has been pleasant.  S3 My decision to use Superstar AI Assistant was a wise one.  S4 I am happy with the results of using Superstar AI Assistant in teaching.  S5 Superstar AI Assistant meets my teaching needs.  S6 Compared with other tools, I feel satisfied with Superstar AI Assistant. | Bhattacherjee (2001); Oliver (1997); Wixom & Todd (2005) | **ECM** |
| **Behavioral Intention (BI)** (6 items) | The degree to which a user intends to continue or increase system use in the future. | BI1 I intend to keep using Superstar AI Assistant in future teaching.  BI2 I will frequently use Superstar AI Assistant in my work.  BI3 I plan to use Superstar AI Assistant whenever possible.  BI4 I will recommend Superstar AI Assistant to colleagues.  BI5 Assuming I have access, I expect to continue using Superstar AI Assistant.  BI6 I prefer Superstar AI Assistant over other teaching platforms. | Venkatesh et al. (2003); Venkatesh & Davis (2000); Bhattacherjee (2001) | **UTAUT / ECM (Behavioral Intention)** |
| **Use Behavior (UB)** (6 items) | The extent of actual system usage in terms of frequency, duration, and integration into work tasks. | UB1 I use Superstar AI Assistant regularly during the semester.  UB2 I use Superstar AI Assistant in most of my classes.  UB3 I spend considerable time using Superstar AI Assistant for course preparation.  UB4 I rely on Superstar AI Assistant for teaching and assessment tasks.  UB5 I have continued to use Superstar AI Assistant after initial adoption.  UB6 My overall level of Superstar AI Assistant use is high. | Venkatesh et al. (2003); Bhattacherjee (2001) | **UTAUT / ECM (Use Behavior)** |
